# Surgical Experience of Transcranial Approaches to Large-to-Giant Pituitary Adenomas in Knosp Grade 4

**DOI:** 10.3389/fendo.2022.857314

**Published:** 2022-05-12

**Authors:** Xiudong Guan, Yangyang Wang, Chengkai Zhang, Shunchang Ma, Wenjianlong Zhou, Guijun Jia, Wang Jia

**Affiliations:** ^1^ Department of Neurosurgery, Beijing Tiantan Hospital, Capital Medical University, Beijing, China; ^2^ Beijing Neurosurgical Institute, Beijing, China

**Keywords:** pituitary macroadenoma, Knosp grade 4, transcranial approach, outcome, frontolateral approach, frontotemporal approach

## Abstract

Pituitary adenomas in Knosp grade 4 are difficult to resect completely and are generally involved in poor prognosis, because of the close relationship between the tumor and internal carotid. In this study, the authors retrospectively reviewed the outcome of different transcranial approaches in the management of large-to-giant pituitary adenomas in Knosp grade 4. A total of 42 patients with large-to-giant pituitary adenomas in Knosp grade 4, who underwent craniotomy in the Pituitary Disease Subdivision, Department of Neurosurgery, Beijing Tiantan Hospital, between March 2012 and March 2015 were included in this study. Clinical characteristics, surgical methods, complications, and outcomes were evaluated. The median age was 45 years (range, 19–73 years old), and 42.9% of the enrolled cases were men. The mean tumor diameter was 43.6 mm, and the mean volume was 30.9 cm^3^. 26 patients underwent the frontolateral approach, while 16 cases accepted the frontotemporal approach. Gross total resection was achieved in 11 patients (26.2%), near total in 26 (61.9%), and subtotal in 5 (11.9%). The adenomas were larger, and the distance of the tumor extending to the lateral skull base was also further in the frontotemporal approach cases. The surgical time was shorter, and the bleeding volume was less in the frontolateral approach cases. Subsellar extension was associated with incomplete resection in pituitary macroadenomas of Knosp grade 4. The craniotomy is still an effective treatment for pituitary macroadenomas in Knosp grade 4.

## Introduction

As one of the most common benign tumors in the brain, pituitary adenoma accounts for 10%–15% of all intracranial tumors (1, 2). However, approximately 20%–55% of pituitary adenomas present an aggressive behavior and invade surrounding structures, such as the third ventricle, cavernous sinus, and sphenoid sinus. According to the Knosp classification, Knosp 3–4 was considered as a cavernous sinus invasion (3). The surgical strategy for pituitary adenomas with the cavernous sinus invasion and large tumor volume is particularly challenging, due to the deep intracranial location and being close to critical neurovascular structures.

The transsphenoidal approach is the preferred treatment in the surgical therapy of pituitary adenoma, which involves fewer complications (4). The improvements in visualization and additional lighting and the application of neuroendoscopy allow neurosurgeons to better distinguish tumor from normal tissue (5). The transsphenoidal approach is the preferred choice for pituitary adenomas with mild cavernous sinus invasion (Knosp grades 1–2), even part of adenomas in Knosp grade 3. However, endoscopic surgery still works less well in large-to-giant adenomas with multilobular configuration and extension beyond the lateral wall of the cavernous sinus, due to a narrow surgical working channel (6). Because of the adjacency of neurovascular structures in the cavernous sinus and the complicated anatomy of the skull base, it is difficult to completely remove the pituitary adenomas in Knosp grade 4 (7, 8). The incomplete resection rate is still up to 50%–65% of pituitary adenomas with the cavernous sinus invasion, even though the extended endoscopy technique has been gradually employed (9–11).

The transcranial approach is still essential for 1%–10% of large-to-giant adenomas with irregular shape and extension into the subfrontal region, retrochiasmatic area, or temporal region (6, 12). Recently, limited articles have addressed the transcranial approach to large-to-giant adenomas in Knosp grade 4.

The present study provides the outcome and complications of 42 patients with large-to-giant pituitary adenomas in Knosp grade 4 treated by the transcranial approach.

## Materials and Methods

### Data Collection

**Figure 1:**
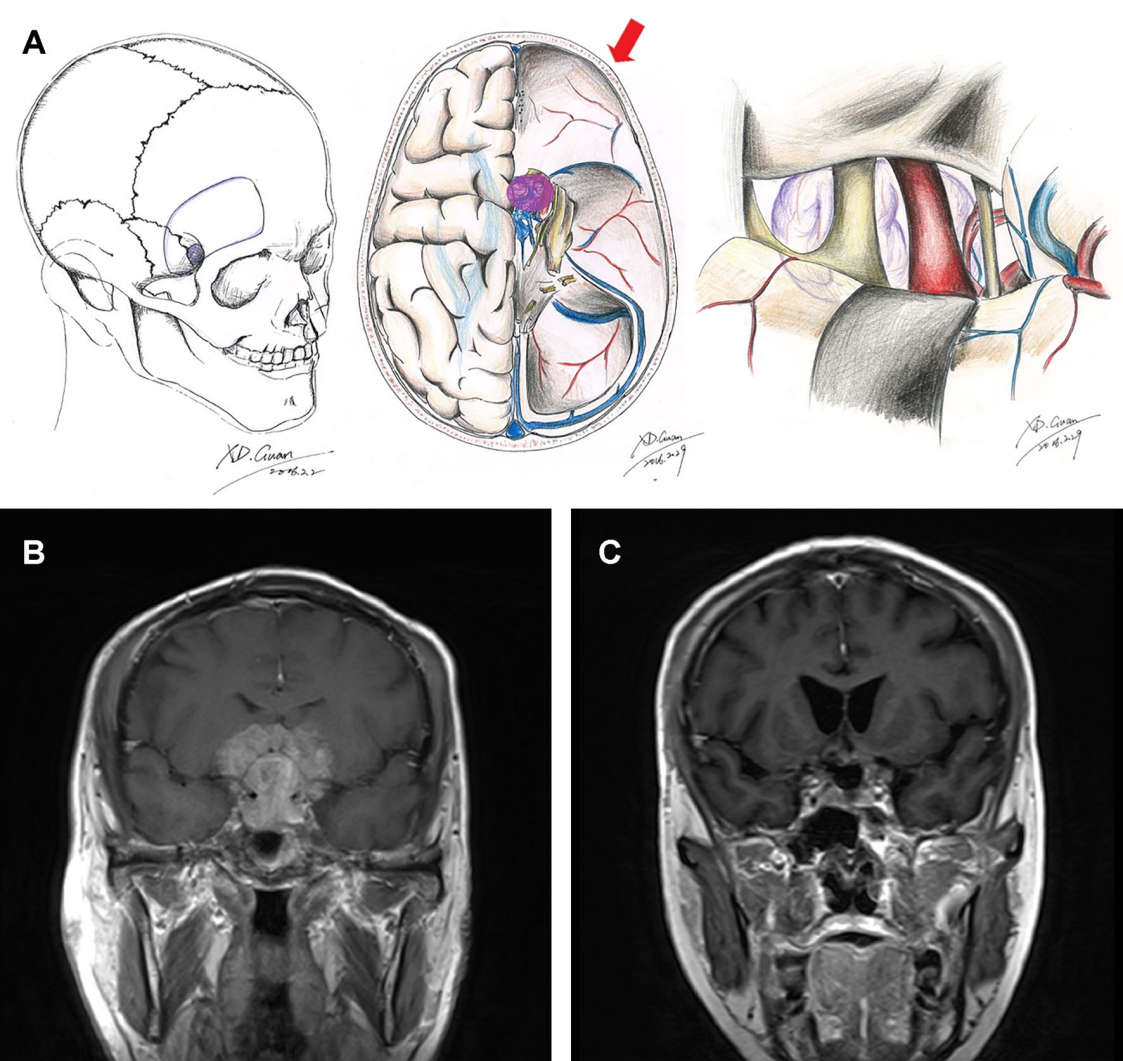
Schematic diagrams and MRI images of frontolateral approach. Schematic diagrams of incision, surgical field, and microanatomy of frontolateral approach **(A)**. Preoperative **(B)** and 3-month postoperative **(C)** coronal enhanced MRI images of a giant pituitary adenoma in Knosp grade 4 that underwent the frontolateral approach.

**Figure 2:**
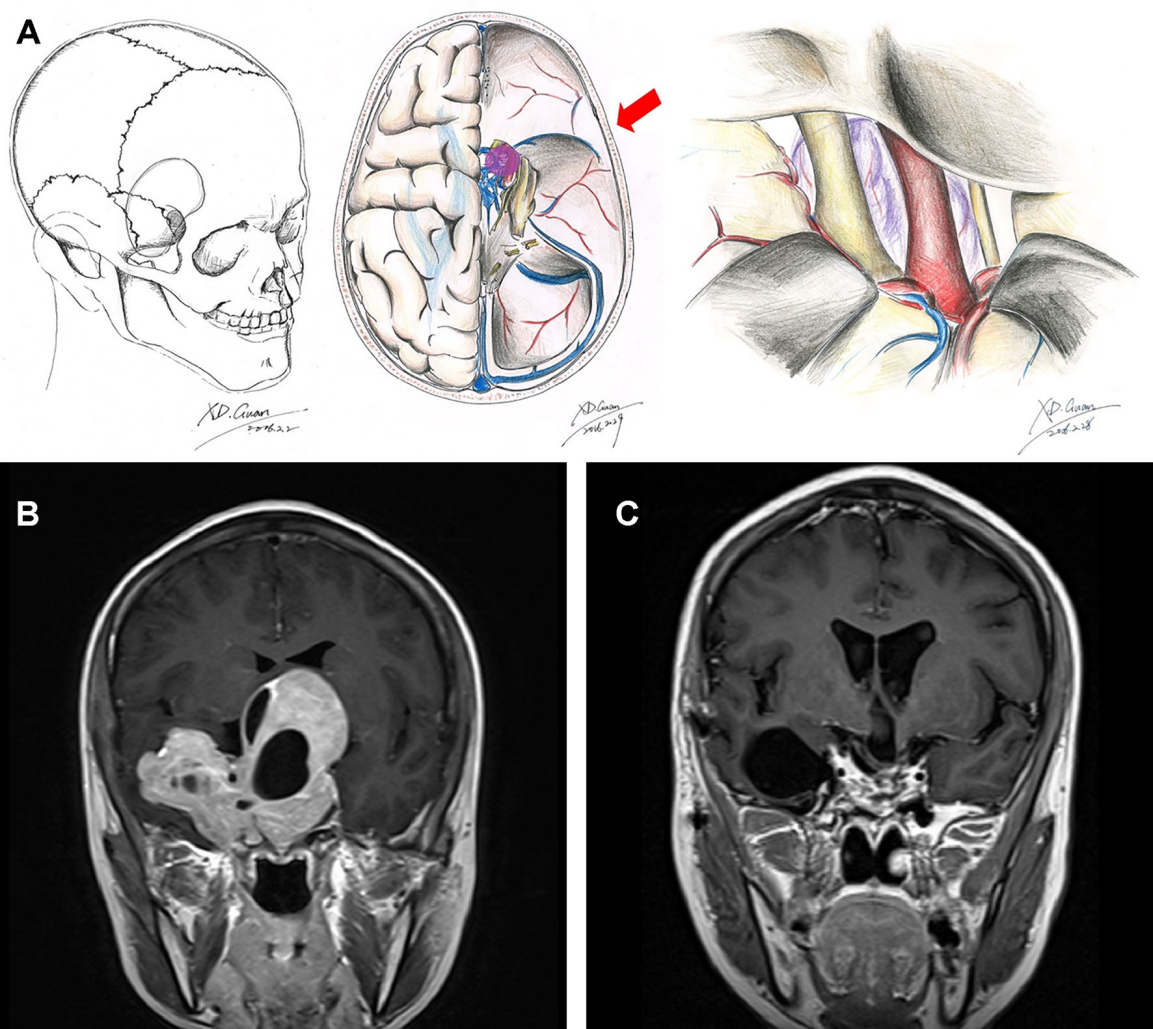
Schematic diagram and MRI images of the frontotemporal approach. Schematic diagrams of incision, surgical field, and microanatomy of frontolateral approach **(A)**. Preoperative **(B)** and 3-month postoperative **(C)** coronal enhanced MRI images of a giant pituitary adenoma in Knosp grade 4 that underwent the frontotemporal approach.

The ethical review committee at the Capital Medical University approved this study. A prospectively acquired database on all patients with pituitary adenoma who underwent surgery in the Pituitary Disease Subdivision, Department of Neurosurgery, Beijing Tiantan Hospital, Capital Medical University, between March 2012 and March 2015 was retrospectively reviewed. All surgeries were performed by the senior authors (WJ and GJ). A total of 625 patients were pathologically confirmed to be pituitary adenoma during this time period. After excluding patients who are <18 years old or underwent transsphenoidal approach surgery, we finally confirmed 42 cases of craniotomy with pituitary adenomas in Knosp grade 4. The surgical approaches were discussed by neurosurgeons in our department and finally determined by two senior neurosurgeons (WJ and GJ). Tumors with mainly suprasellar invasion or a dumbbell shape tended to be treated with the frontolateral approach (**Figures 1A–C**). Pituitary adenoma in Knosp grade 4 with a large eccentric extension into the middle or posterior cranial fossa or temporal lobe tended to be dealt with using the frontotemporal approach (**Figures 2A–C**).

### Radiological Findings

Magnetic resonance images (MRI) were acquired in each patient using a standard 3.0-T scanner preoperatively and postoperatively. The neurosurgeons interpreted the pre- and postsurgical MRI findings based on the T1-weighted coronal slices with and without contrast enhancement. Parasellar extension was evaluated by the Knosp grading scale (3) (grade 3 and grade 4). Suprasellar extension was identified according to the Wilson–Hardy grade (2) (grade C and grade D). Subsellar extension was estimated by the results of computed tomography (CT) combined with the MRI findings and intraoperative observation. The invasion and resection status of pituitary adenomas was respectively assessed by WJ, XG, and WZ, who provided independent evaluations in an attempt to decrease the reporting bias. To quantitatively define the tumor size, the maximum diameter of the tumor was measured based on the T1-weighted coronal slices with and without contrast enhancement from the axial, sagittal, and coronal images. The tumor volume was calculated according to the formula: V = abcπ/6 (a: length; b: width; c height).

### Endocrinological and Ophthalmological Evaluations

Pituitary hormone measurements, visual field tests, and visual acuity tests were performed in all patients preoperatively and 3 months postoperatively. The hormone panels included prolactin, GH, IGF-1, cortisol, ACTH, LH/FSH, progesterone, estradiol, testosterone, FT3, TT3, FT4, TT4, and TSH levels. Hormone remission was defined according to the following criteria: for patients with prolactinoma, serum PRL <20 ng/ml in women or <15 ng/ml in men (13); for patients with acromegaly, normalized age-adjusted insulin-like growth factor-1 level (IGF-1) and GH random level <1 ng/ml, or an oral glucose tolerance test <0.4 ng/ml (14, 15); and for patients with Cushing’s syndrome, morning serum cortisol values <5 μg/dl or urine free cortisol <10–20 g/day (16). Male patients with reduced testosterone or female patients with low FSH levels were considered as hypogonadism. Patients with reduced fT4 and/or elevated TSH were identified as hypothyroidism. Patients with low cortisol levels were defined as hypocortisolism. Patients with low levels of all pituitary hormones were considered as panhypopituitarism.

### Tumor Subtypes

Tumors were classified according to the hormone level and pathological diagnosis (17, 18). According to hormone level, tumors were classified into functioning adenomas and non-functioning adenomas. According to the histopathologic findings, non-functioning adenomas were further divided into null cell adenomas and silent pituitary adenomas (including PRL positive, GH positive, ACTH positive, FSH/LH positive, TSH positive, or plurihormonal positive).

### Follow-Up

The MRI, neurological, and endocrinological evaluations were repeated at 3, 6, and 12 months followed by per year after surgery.

### Statistics Analysis

Statistics analysis was performed using IBM SPSS Statistics software (version 24.0, Armonk, NY: IBM Corp). Figures were made by GraphPad Prism 7.0 for Mac OS (GraphPad Software, La Jolla CA, USA). The chi-square test was used to compare categorical data, whereas an unpaired t-test was used to compare subgroup means. The Mann–Whitney U test was performed to compare the postoperative visual acuity, visual field, and resection rate between two surgical approaches. Univariable logistic regressions were performed to analyze predictors of gross total resection. A two-tailed *P*-value < 0.05 was considered statistically significant.

## Results

### Clinical Features

A total of 42 patients (18 men; 42.9%) fulfilled the criteria for this study. The median age was 44 years (range, 19–73 years old). As shown in **Table 1**, the primary presenting symptom was progressive visual loss (83.3%), followed by headache (35.7%) and endocrinopathy (23.8%). The majority of patients were diagnosed with non-functioning pituitary adenomas (36/42, 85.7%). 6 patients were diagnosed with functioning pituitary adenomas, including 2 PRL-secreting adenomas, 3 GH-secreting adenomas, and 1 ACTH-secreting adenoma. 6 patients had recurrent tumors after prior microscopic transsphenoidal or transcranial surgery. Hemianopsia was observed in 31 patients (73.8%) *via* visual field testing. 3 patients (7.1%) had other cranial nerve palsies. 44.4% (8/18) of men had hypogonadism, while 37.5% (9/24) of women presented with hypogonadism. 5 patients had hypothyroidism, 5 patients had hypocortisolism, and 3 patients presented panhypopituitarism (**Table 1**).

**Table 1:** Clinical characteristics of 42 patients with large to giant pituitary adenomas in Knosp grade 4.

Clinical characteristics	Value
**Sex, male/female**	18/24
**Age (median ± SD [range]) (years)**	44 ± 13 (19–73)
**Presenting symptoms, n (%)**	
Impaired visual acuity	35 (83.3)
Headache	15 (35.7)
Endocrinopathy	10 (23.8)
Incidental	1 (2.4)
Recurrence	6 (14.3)
**Vision, n (%)**	
Hemianopsia	31 (73.8)
Cranial nerve deficit	3 (7.1)
Other	5 (11.9)
Normal	4 (9.5)
**Endocrinological types, n (%)**	
Non-functioning adenomas	36 (85.7)
Functioning adenomas	6 (14.3)
PRL	2 (4.8)
GH	3 (7.1)
ACTH	1 (2.4)
**Endocrine function, n (%)**	
Hypogonadism, male	8 (44.4 *^a^*)
Hypogonadism, female	9 (37.5 *^b^*)
Hypothyroidism	5 (11.9)
Hypocortisolism	5 (11.9)
Panhypopituitarism	3 (7.1)

a Number of hypogonadism in male/total number of male patients.

b Number of hypogonadism in female/total number of female patients.

Preoperative MRI findings demonstrated macroadenomas (>10 mm) in all patients. The maximum diameter of the tumor ranged from 25 to 76 mm (mean, 43.6 mm; SD, 11.9 mm). The maximal diameters in most adenomas (61.9%) were more than 40 mm, followed by 30 to 40 mm (26.2%) and 20 to 30 mm (11.9%). The mean approximated volume was 30.9 ± 27.8 cm^3^. Besides, 76.2% of adenomas were more than 10 cm^3^. Moreover, 21.4% of adenomas were the bilateral parasellar invasion. All adenomas were accompanied by suprasellar extension and compression of the optic chiasm. 40.5% of adenomas were exhibited with subsellar extension. 14.3% of tumors had a cystic formation (**Table 2**).

**Table 2:** Imaging characteristics of large to giant pituitary adenomas in Knosp grade 4.

Tumor characteristic	Value
**Tumor diameter, mean ± SD, mm**	43.6 ± 11.9
≤30 mm, >20 mm, n (%)	5 (11.9)
≤40 mm, >30 mm, n (%)	11 (26.2)
>40 **mm**, n (%)	26 (61.9)
**Tumor volume, mean ± SD, cm^3^**	30.9 ± 27.8
≤10 cm^3^, n (%)	10 (23.8)
>10 cm^3^, n (%)	32 (76.2)
**Chiasm compression, n (%)**	42 (100)
**Parasellar invasion, n (%)**	
Unilateral	33 (78.6)
Bilateral	9 (21.4)
**Invasive localization, n (%)**	
With suprasellar extension	42 (100)
With subsellar extension	17 (40.5)
**Cystic formation, n (%)**	6 (14.3)

### Surgical Approach

In this study, all patients underwent craniotomy. Surgical data were summarized as shown in **Table 3**. Among these, 61.9% of tumors were removed by the frontolateral approach (FL), and the others were *via* the frontotemporal approach (FT). The mean surgical time was 286 ± 83 min, while the mean amount of bleeding volume was 745 ± 696 ml. Near-total resection (61.9%) was the most, followed by gross total resection (26.2%) and subtotal resection (11.9%).

**Table 3:** Surgery and extent of resection.

Characteristic	Value
**Surgical procedures, n (%)**	
Frontolateral approach	26 (61.9)
Frontotemporal approach	16 (38.1)
**Surgical time, mean ± SD, min**	286 ± 83
**Bleeding volume, mean ± SD, ml**	745 ± 696
**Extent of resection, n (%)**	
Gross total	11 (26.2)
Near total	26 (61.9)
Subtotal	5 (11.9)

**Figure 3:**
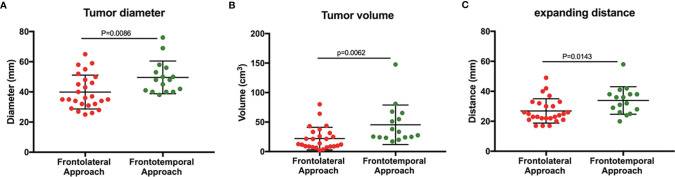
Tumor size in the patients who underwent different surgical approaches. **(A)** The maximum diameter of tumors in two groups. **(B)** The tumor volume in two groups. **(C)** The distance of tumors expanding to the lateral skull base (Student t-test, P < 0.05).

In our study, we found that the maximum diameter of tumors removed by the frontolateral approach (mean ± SD, 39.9 ± 11.8 mm) was significantly smaller than that of tumors resected by the frontotemporal approach (mean ± SD, 49.6 ± 10.2 mm) (*P* = 0.009) (**Figure 3A**). Besides, the tumor volumes in the FL group were smaller compared with the FT group (mean ± SD, 21.9 ± 19.1 cm^3^ vs. 45.4 ± 33.6 cm^3^, *P* = 0.006), (**Figure 3B**). According to the classification of tumor size, the number of giant adenomas in the FT group was also higher than that in the FL group (*P* = 0.003, χ^2^ = 9.040) (**Table 4**). Furthermore, we measured the distance from the tumor border in the lateral skull base to the tumor center. The expanding distance in the FL (mean ± SD, 26.9 ± 1.6 mm) was also shorter compared with the FT group (mean ± SD, 33.9 ± 2.3 mm) (*P* = 0.014) (**Figure 3C**). Due to the invasion of tumors, the number of patients with subsellar extension in the FL group was more than in the FT group (*P* = 0.023, χ^2^ = 5.203) (**Table 4**). However, there was no significance between the two groups in the unilateral or bilateral parasellar invasion. Two groups also had no difference in the status of cystic formation and endocrinological types (**Table 4**).

**Table 4:** Surgical approach based on preoperative imaging characteristic.

	Frontolateral approach (N = 26) n (%)	Frontotemporal approach (N = 16) n (%)	*P* value
**Tumor diameter**			
Macroadenoma (≤40 mm, >10 mm)	15 (57.7)	1 (6.3)	0.003 *^a^*
Giant adenoma (>40 mm)	11 (42.3)	15 (93.8)	
**Tumor volume**			
Macroadenoma (≤10 cm^3^)	10 (38.5)	0 (0.0)	0.007 *^b^*
Giant adenoma (>10 cm^3^)	16 (61.5)	16 (100)	
**Endocrinological types**			
Non-function adenomas	24 (92.3)	12 (75)	0.27 *^a^*
Functioning adenomas	2 (7.7)	4 (25)	
**Parasellar invasion**			
Unilateral	23 (88.5)	10 (62.5)	0.109 *^a^*
Bilateral	3 (11.5)	6 (37.5)	
**With subsellar extension**	7 (26.9)	10 (62.5)	0.023 *^c^*
**Cystic formation**	3 (11.5)	3 (18.8)	0.846 *^c^*

a Continuity correction.

b Fisher’s exact test.

c Pearson chi-square test.

### Clinical Outcome and Complications

According to the postoperative histopathological testing, there were 22 null cell adenomas, 12 silent adenomas (including 1 PRL-positive, 1 GH-positive, 1 ATCH-positive, 5 FSH/LH-positive, 2 TSH-positive, and 4 plurihormonal-positive) among non-functioning adenomas. Among functioning adenomas, there were 2 PRL-positive adenomas, 1 GH-positive adenoma, 1 ACTH-positive adenoma, and 2 plurihormonal positive adenomas (**Table 5**). In functioning adenomas, the remission ratio was 1/2 in PRL adenomas, 0/3 in GH adenomas, and 1/1 in ACTH adenomas (**Table 5**).

**Table 5:** Clinical outcome and complications.

Characteristic	Value
**Histopathology, n (%)**	
Non-functioning adenomas	36 (85.7)
Null cell	22 (52.4)
PRL positive	1 (2.4)
GH positive	1 (2.4)
ACTH positive	1 (2.4)
FSH/LH positive	5 (11.9)
TSH positive	2 (4.8)
Plurihormonal positive	4 (9.5)
Functioning adenomas	6 (14.3)
PRL positive	2 (4.8)
GH positive	1 (2.3)
ACTH positive	1 (2.3)
Plurihormonal positive	2 (4.8)
**Hormone remission, remission/total**	
PRL	1/2
GH	0/3
ACTH	1/1
**Visual acuity**	
Improve	11 (26.2)
No change	23 (54.8)
Worsen	8 (19.0)
**Visual field**	
Improve	7 (16.7)
No change	31 (73.8)
Worsen	4 (9.5)
**Complications**	
Hemorrhage	2 (4.8)
Infection	9 (21.4)
Nerve palsies	16 (38.1)
Electrolyte disorder	20 (47.6)

No serious complications such as perioperative death or internal carotid artery injury occurred in the cohort. According to postoperative visual acuity testing, 11 of 35 patients with impaired visual acuity exhibited visual development after surgery, while the visual acuity of 8 patients decreased (**Table 5**). Meanwhile, 7 of 31 patients with hemianopsia revealed improvement in the visual field (**Table 5**). However, the defect of the visual field was worsened in 4 patients. Meanwhile, hemorrhage happened in two cases after surgery. 21.4% of patients suffered from postoperative infection. 16 patients presented nerve palsies, and electrolyte disturbance was shown in 20 cases (**Table 5**).

There was no difference in the extent of resection, change of visual acuity, and visual field between the two approaches. The hormone remission ratios of the FL and FT groups were 1/4 and 1/2, respectively. It was a trend that hemorrhage, infection, nerve palsies, or electrolyte disorder were more prevalent in the FT group than in the FL group, although there was no significant difference in the outcome between the two groups (**Table 6**).

**Table 6:** Outcome and complications between different surgical approach.

	Frontolateral approach (N = 26)	Frontotemporal approach (N = 16)	*P* value
**Extent of resection, n (%)**			
Gross total	8 (30.8)	3 (18.8)	0.102 *^a^*
Near total (>90%)	17 (65.4)	9 (56.3)	
Subtotal	1 (3.8)	4 (25.0)	
**Outcome, n (%)**			
**Visual acuity**			
Improve	7 (26.9)	4 (25.0)	0.943 *^a^*
No change	14 (53.8)	9 (56.3)	
Worsen	5 (19.2)	3 (18.8)	
**Visual field, n (%)**			
Improve	5 (19.2)	2 (12.5)	0.480 *^a^*
No change	19 (73.1)	12 (75.0)	
Worsen	2 (7.7)	2 (12.5)	
**Hormone remission, remission/total**	1/4	1/2	
**Complications, n (%)**			
Hemorrhage	1 (3.8)	1 (6.3)	
Infection	5 (19.2)	4 (25.0)	0.956 *^b^*
Nerve palsies	9 (34.6)	7 (43.8)	0.554 *^c^*
Electrolyte disorder	10 (38.5)	10 (62.5)	0.130 *^c^*

a Mann–Whitney U test.

b Continuity correction.

c Pearson chi-square test.

Preoperative patients’ clinical and radiological characteristics were analyzed to investigate the ability to predict gross total resection (**Table 7**). The subsellar extension was a potential unfavorable factor for gross total resection of pituitary macroadenomas in Knosp grade 4 (P = 0.033, OR 10.667, 95% CI 1.214–93.699). However, tumor volume and expanding distance showed no significance, although it was a trend that large volume or expanding distance contributed to the incomplete resection. Sex, age, cystic formation, and surgical approach were not significant predictors for gross total resection.

**Table 7:** Univariable logistic regression analyses of predictors for gross total resection.

Characteristics	OR	95% CI	*P* value
Sex (male)	2.500	0.557–11.230	0.232
Age (≥45 years)	0.889	0.224–3.534	0.867
Max diameter (>40 mm)	2.520	0.618–10.276	0.197
Tumor volume (>10 cm^3^)	4.333	0.843–19.905	0.059
Expanding (≥27 mm)	3.692	0.819–16.656	0.089
Subsellar extension (with)	10.667	1.214–93.699	0.033
Cystic formation (with)	0.667	0.104–4.272	0.669
Surgical approach (frontotemporal)	1.926	0.427–8.688	0.394

### Follow-Up

36 patients (85.7%) were followed up by 15–46 months after surgery (median 30 months). Among them, 13 patients accepted adjuvant gamma knife treatment. Moreover, 3 of 36 patients presented recurrence. 18 patients presented the progression of residual tumors. Meanwhile, the residual adenomas were stable in 13 cases. The visual acuity of 4 patients was improved during the follow-up period. However, one patient’s visual acuity deteriorated. 4 patients still exhibited endocrine disorder.

## Discussion

As the most common lesion in the saddle area, some pituitary adenomas exhibit infiltration into the cavernous sinus structure and a close relationship with the internal carotid. Besides, neurosurgeons are in deep trouble with invasive pituitary macroadenomas as the result of low total removal rate, serious complaints, and high recurrent rate. In this study, we retrospectively reviewed 42 cases with pituitary macroadenomas in Knosp grade 4 who underwent craniotomies. A total of 26 patients underwent frontolateral approaches to remove the tumor, and 16 macroadenomas were excised by frontotemporal approaches. The gross total resection rate, near-total resection rate, and subtotal resection rate were 26.2%,11.9%, and 61.9%, respectively. In the surgical findings, the surgical time was shorter, and the bleeding volume was less through the frontolateral approach than through the frontotemporal approach. However, there was no statistical significance in postoperative complaints. The subsellar invasion was a predictor for incomplete resection.

Patients with pituitary macroadenomas generally have a poor prognosis, such as low gross total resection (GTR) rate, high rate of complications, and recurrence (19–24). Besides, the surgery for parasellar invasive adenomas, especially in Knosp grade 4, is a hot topic in neurosurgeons. As a result, our study focused on the outcome of patients with pituitary macroadenomas in Knosp grade 4. It is a common view that transsphenoidal surgery is the first-line treatment for patients with pituitary adenomas. With advances in technique and experience, mounting evidence suggests that endoscopic transsphenoidal surgery is a substitute for microscopic surgery for properly selected patients with pituitary macroadenomas (6, 25–27). With the development of endoscopic techniques, the endoscopic transsphenoidal approach has become the first-line treatment for the majority of pituitary adenomas. The proportion of patients undergoing the endoscopic transsphenoidal approach gradually increased (28). However, the endoscopic transsphenoidal approach also has some limitations for pituitary adenomas with parasellar invasion or Knosp grade 4. Previous retrospective studies has shown that the GTR rates for tumor extended into the anterior fossa, middle fossa, and posterior fossa were only 7.7%, 19.6%, and 14.3%, respectively (6). For giant pituitary adenomas that dealt with the transsphenoidal endoscopic approach, the GTR rate for rounded, dumbbell, and multilobular tumors was 46.7%–64%, 33.3%–46%, and only 6.1%–8%, respectively (6, 29). For patients with partial resection and intracranial remnant, transcranial reoperation was usually considered (29). In recent years, the indications of the endoscopic transsphenoidal approach gradually became broader. The extended endoscopic transsphenoidal approach can also be applied in selected pituitary adenomas in Knosp grade 4. Although a cohort of pituitary adenomas in Knosp grade 4 reached 70.6% (72/102) after aggressive endoscopic transsphenoidal surgery, life-threating internal carotid artery (ICA) injury occurred in 2 cases (30).

Providing a more panoramic visualization and wider corridor compared with microscopic surgery, extended endoscopic endonasal transsphenoidal surgery does not take the invasion of the medial wall of the cavernous sinus as a contraindication of tumor resection (6, 27, 31). However, the invasion of the lateral wall of the cavernous sinus, even the extension to the temporal lobe, is labeled as a limitation of gross total resection (6). The rate of GTR was 0% to 8% in the cases of macro or giant adenomas with Knosp grade 4 invasion (6, 27, 32, 33). Also, another reason that extensive macro or giant adenomas can be resected through craniotomy is that continuous stable displacement of the neurovascular structures, due to the slow progression of the tumor over decades, provides a potentially large preformed space for resection corridors. The exposure in the frontolateral craniotomy from the contralateral optic nerve to the ipsilateral oculomotor nerve supplies neurosurgeons variety of corridors to resect tumor through the classic subchiasmina, opticocarotid, carotid-oculomotor, and translamina terminalis pathways, allowing resection of deeply located parts of the tumor (**Figure 1A**). These kinds of multiple corridors avoid the collapse of the tumor bed after the previous steps of resection and maintains the exposure of tumor mass, allowing access to the deeper parts of the tumor (34). As a result of the exposure in this approach including the corridors in the subfrontal approach and frontotemporal approach, we do not require a larger opening at the skull surface to remove a deep lesion. Besides, the frontolateral approach could reduce the incidence of damage to the branch of the facial nerve, supraorbital nerve, and temporal muscle and protect the frontal lobe. However, using this approach is difficult to overcome parts of the lesion extending to the third ventricle, which is not available to get a good surgery field, and is difficult to protect the frontal lobe.

Provided drilling the sphenoid ridge as far down as the superior orbital fissure, the frontotemporal (pterional) approach allows neurosurgeons to reach the tumor *via* the lateral fissure (**Figure 2A**), which is the natural space between two lobes. The exposure in the frontotemporal approach from the ipsilateral optic nerve to the oculomotor supplies opticocarotid and carotid-oculomotor pathways to remove the deeply located parts of the tumor (**Figure 2A**). Besides, this approach also provides a chance to remove the parts of the tumor invading into the cavernous sinus structure *via* opening the lateral wall of the cavernous sinus. Nevertheless, this surgical approach requires consideration of protecting the temporal branches of the facial nerve (35). Damaging them in the surgery may lead to the paralysis of the frontalis, orbicularis oculi, and corrugator supercilii muscles.

In our study, GTR, evaluated by no residual lesion on the postoperative MRI, was achieved in 26.2% of patients. In addition, the rate of near-total resection was 61.9%. These rates of resection correlate well with some other reports, whose rates are less than 10% (7, 8, 24, 36). The GTR of patients, who underwent the frontolateral approach, was not significantly different from the frontotemporal approach, although a trend toward the GTR in the frontolateral approach was better. Compared with the FL group, the tumor size in the FT group was larger. More importantly, the tumor treated by the FT approach extended further to the lateral skull base than those treated by the TL approach. We chose the frontotemporal approach to protect the frontal lobe. However, the frontotemporal approach was more complex than in the frontolateral craniotomy, leading to a long surgical time and more bleeding volume. Furthermore, the extent of resection and complications did not reach statistical significance between the two surgical approaches.

The complete resection contributes to a decrease in the risk of recurrence and an increase in the chance of endocrinological remission. Although the endoscopic transsphenoidal approach is viewed as the first-line treatment, transcranial surgery still has the advantage of removing the pituitary adenomas with parasellar invasion, especially Knosp grade 4. In this study, we analyzed the preoperative characters of the tumors to predict the extent of resection. Our experience showed that subsellar extension increased the risk of the residual lesion for macroadenomas in Knosp grade 4 when treated by the transcranial approach. In such cases, staging surgery would be a better choice to increase the chance of cure.

There were also some limitations in this study. This study was a retrospective research, and risks of selection bias existed. The operations performed in this series were designed by neurosurgeons in a single center, after a conference discussion according to the preoperative examination. Therefore, the choice of the surgical approach was subjective to some extent. We focused on the transcranial approaches for Knosp grade 4 adenoma. However, we did not discuss combined transsphenoidal and transcranial approaches.

## Conclusion

Although the endoscopic transsphenoidal approach is the first-line treatment for pituitary adenoma, the transcranial approach acts as an alternative and still has its value for pituitary adenoma in Knosp grade 4. In the present study, 26.2% (11/42) pituitary adenoma in Knosp grade 4 achieved gross total resection after the transcranial approach. Compared with the frontolateral approach, the frontotemporal approach was more appropriate for tumors in large volume and further extended into the lateral skull base. At the same time, the frontotemporal approach was associated with longer surgical time and more bleeding volume. Subsellar extension was associated with incomplete resection in pituitary macroadenomas of Knosp grade 4. Our analysis summarized the risks and benefits of common transcranial approaches and provided evidence for the design of surgical procedures for large to giant pituitary adenomas in Knosp grade 4.

## Data Availability Statement

The raw data supporting the conclusions of this article will be made available by the authors, without undue reservation.

## Ethics Statement

The studies involving human participants were reviewed and approved by the ethical review committee at the Capital Medical University. Written informed consent for participation was not required for this study in accordance with the national legislation and the institutional requirements.

## Author Contributions

WJ and XG designed the study. XG, YW, CZ, and WZ acquired the data. XG, CZ, and SM analyzed and interpreted the data. WJ and GJ performed surgery on patients. XG and YW wrote the first draft. WJ was responsible for the integrity and accuracy of the data and was the supervisor. All authors contributed to the article and approved the submitted version.

## Funding

This work was supported by the Capital Health Research and Development of Special (2014–2–1072) and the Beijing Municipal Natural Science Foundation (7142054).

## Conflict of Interest

The authors declare that the research was conducted in the absence of any commercial or financial relationships that could be construed as a potential conflict of interest.

## Publisher’s Note

All claims expressed in this article are solely those of the authors and do not necessarily represent those of their affiliated organizations, or those of the publisher, the editors and the reviewers. Any product that may be evaluated in this article, or claim that may be made by its manufacturer, is not guaranteed or endorsed by the publisher.

## References

[1] Kovacs K Horvath E. Pathology of Pituitary Tumors. Endocrinol Metab Clin North Am (1987) 16(3):529–51. doi: 10.1016/S0889-8529(18)30463-8 3319594

[2] Wilson CB. A Decade of Pituitary Microsurgery. The Herbert Olivecrona Lecture. J Neurosurg (1984) 61(5):814–33. doi: 10.3171/jns.1984.61.5.0814 6092567

[3] Knosp E Steiner E Kitz K Matula C. Pituitary Adenomas With Invasion of the Cavernous Sinus Space: A Magnetic Resonance Imaging Classification Compared With Surgical Findings. Neurosurgery (1993) 33(4):610–7; discussion 7-8. doi: 10.1227/00006123-199310000-00008 8232800

[4] Fahlbusch R Buchfelder M. Transsphenoidal Surgery of Parasellar Pituitary Adenomas. Acta Neurochirurgica (1988) 92(1-4):93–9. doi: 10.1007/BF01401978 3407479

[5] Eseonu CI ReFaey K Rincon-Torroella J Garcia O Wand GS Salvatori R. Endoscopic Versus Microscopic Transsphenoidal Approach for Pituitary Adenomas: Comparison of Outcomes During the Transition of Methods of a Single-Surgeon. World Neurosurg (2016) 97:317–25. doi: 10.1016/j.wneu.2016.09.120 27742515

[6] Koutourousiou M Gardner PA Fernandez-Miranda JC Paluzzi A Wang EW Snyderman CH. Endoscopic Endonasal Surgery for Giant Pituitary Adenomas: Advantages and Limitations. J Neurosurg (2013) 118(3):621–31. doi: 10.3171/2012.11.JNS121190 23289816

[7] Nishioka H Fukuhara N Horiguchi K Yamada S. Aggressive Transsphenoidal Resection of Tumors Invading the Cavernous Sinus in Patients With Acromegaly: Predictive Factors, Strategies, and Outcomes. J Neurosurg (2014) 121(3):505–10. doi: 10.3171/2014.3.JNS132214 25014437

[8] Micko AS Wohrer A Wolfsberger S Knosp E. Invasion of the Cavernous Sinus Space in Pituitary Adenomas: Endoscopic Verification and its Correlation With an MRI-Based Classification. J Neurosurg (2015) 122(4):803–11. doi: 10.3171/2014.12.jns141083 25658782

[9] Dhandapani S Singh H Negm HM Cohen S Anand VK Schwartz TH. Cavernous Sinus Invasion in Pituitary Adenomas: Systematic Review and Pooled Data Meta-Analysis of Radiologic Criteria and Comparison of Endoscopic and Microscopic Surgery. World Neurosurg (2016) 96:36–46. doi: 10.1016/j.wneu.2016.08.088 27591098

[10] Paluzzi A Fernandez-Miranda JC Tonya Stefko S Challinor S Snyderman CH Gardner PA. Endoscopic Endonasal Approach for Pituitary Adenomas: A Series of 555 Patients. Pituitary (2014) 17(4):307–19. doi: 10.1007/s11102-013-0502-4 23907570

[11] Dehdashti AR Ganna A Karabatsou K Gentili F. Pure Endoscopic Endonasal Approach for Pituitary Adenomas: Early Surgical Results in 200 Patients and Comparison With Previous Microsurgical Series. Neurosurgery (2008) 62(5):1006–15; discussion 15-7. doi: 10.1227/01.neu.0000325862.83961.12 18580798

[12] Pratheesh R Rajaratnam S Prabhu K Mani SE Chacko G Chacko AG. The Current Role of Transcranial Surgery in the Management of Pituitary Adenomas. Pituitary (2013) 16(4):419–34. doi: 10.1007/s11102-012-0439-z 23076713

[13] Casanueva F Molitch M Schlechte J Abs R Bonert V Bronstein M. Guidelines of the Pituitary Society for the Diagnosis and Management of Prolactinomas. Clin Endocrinol (2006) 65(2):265–73. doi: 10.1111/j.1365-2265.2006.02562.x 16886971

[14] Katznelson L Laws E Melmed S Molitch M Murad M Utz A. Acromegaly: An Endocrine Society Clinical Practice Guideline. J Clin Endocrinol Metab (2014) 99(11):3933–51. doi: 10.1210/jc.2014-2700 25356808

[15] Giustina A Chanson P Bronstein M Klibanski A Lamberts S Casanueva F. A Consensus on Criteria for Cure of Acromegaly. J Clin Endocrinol Metab (2010) 95(7):3141–8. doi: 10.1210/jc.2009-2670 20410227

[16] Nieman L Biller B Findling J Murad M Newell-Price J Savage M. Treatment of Cushing’s Syndrome: An Endocrine Society Clinical Practice Guideline. J Clin Endocrinol Metab (2015) 100(8):2807–31. doi: 10.1210/jc.2015-1818 PMC4525003 26222757

[17] Lim C Korbonits M. Update on the Clinicopathology of Pituitary Adenomas. Endocrine Pract (2018) 24(5):473–88. doi: 10.4158/ep-2018-0034 29498920

[18] Drummond J Roncaroli F Grossman A Korbonits M. Clinical and Pathological Aspects of Silent Pituitary Adenomas. J Clin Endocrinol Metab (2019) 104(7):2473–89. doi: 10.1210/jc.2018-00688 PMC6517166 30020466

[19] Przybylowski CJ Dallapiazza RF Williams BJ Pomeraniec IJ Xu Z Payne SC. Primary Versus Revision Transsphenoidal Resection for Nonfunctioning Pituitary Macroadenomas: Matched Cohort Study. J Neurosurg (2017) 126(3):889–96. doi: 10.3171/2016.3.jns152735 27203142

[20] Mehta GU Oldfield EH. Prevention of Intraoperative Cerebrospinal Fluid Leaks by Lumbar Cerebrospinal Fluid Drainage During Surgery for Pituitary Macroadenomas. J Neurosurg (2012) 116(6):1299–303. doi: 10.3171/2012.3.jns112160 22482793

[21] Magro E Graillon T Lassave J Castinetti F Boissonneau S Tabouret E. Complications Related to the Endoscopic Endonasal Transsphenoidal Approach for Nonfunctioning Pituitary Macroadenomas in 300 Consecutive Patients. World Neurosurg (2016) 89:442–53. doi: 10.1016/j.wneu.2016.02.059 26902781

[22] Hoang N Tran DK Herde R Couldwell GC Osborn AG Couldwell WT. Pituitary Macroadenomas With Oculomotor Cistern Extension and Tracking: Implications for Surgical Management. J Neurosurg (2016) 125(2):315–22. doi: 10.3171/2015.5.jns15107 26566201

[23] Dallapiazza R Bond AE Grober Y Louis RG Payne SC Oldfield EH. Retrospective Analysis of a Concurrent Series of Microscopic Versus Endoscopic Transsphenoidal Surgeries for Knosp Grades 0-2 Nonfunctioning Pituitary Macroadenomas at a Single Institution. J Neurosurg (2014) 121(3):511–7. doi: 10.3171/2014.6.jns131321 24995783

[24] Chabot JD Chakraborty S Imbarrato G Dehdashti AR. Evaluation of Outcomes After Endoscopic Endonasal Surgery for Large and Giant Pituitary Macroadenoma: A Retrospective Review of 39 Consecutive Patients. World Neurosurg (2015) 84(4):978–88. doi: 10.1016/j.wneu.2015.06.007 26074433

[25] Nakao N Itakura T. Surgical Outcome of the Endoscopic Endonasal Approach for non-Functioning Giant Pituitary Adenoma. J Clin Neurosci (2011) 18(1):71–5. doi: 10.1016/j.jocn.2010.04.049 20851609

[26] Cusimano MD Kan P Nassiri F Anderson J Goguen J Vanek I. Outcomes of Surgically Treated Giant Pituitary Tumours. Can J Neurol Sci Le J Canadien Des Sci Neurol (2012) 39(4):446–57. doi: 10.1017/S0317167100013950 22728851

[27] Juraschka K Khan OH Godoy BL Monsalves E Kilian A Krischek B. Endoscopic Endonasal Transsphenoidal Approach to Large and Giant Pituitary Adenomas: Institutional Experience and Predictors of Extent of Resection. J Neurosurg (2014) 121(1):75–83. doi: 10.3171/2014.3.JNS131679 24785323

[28] Crowther S Rushworth R Rankin W Falhammar H Phillips L Torpy D. Trends in Surgery, Hospital Admissions and Imaging for Pituitary Adenomas in Australia. Endocrine (2018) 59(2):373–82. doi: 10.1007/s12020-017-1457-4 29103185

[29] Micko A Agam M Brunswick A Strickland B Rutkowski M Carmichael J. Treatment Strategies for Giant Pituitary Adenomas in the Era of Endoscopic Transsphenoidal Surgery: A Multicenter Series. J Neurosurg (2022) 136(3):776–85. doi: 10.3171/2021.1.Jns203982 34388714

[30] Ouyang T Zhang N Xie S Tang B Li J Xiao L. Outcomes and Complications of Aggressive Resection Strategy for Pituitary Adenomas in Knosp Grade 4 With Transsphenoidal Endoscopy. Front Oncol (2021) 11:693063. doi: 10.3389/fonc.2021.693063 34235083 PMC8255811

[31] Di Maio S Cavallo LM Esposito F Stagno V Corriero OV Cappabianca P. Extended Endoscopic Endonasal Approach for Selected Pituitary Adenomas: Early Experience. J Neurosurg (2011) 114(2):345–53. doi: 10.3171/2010.9.jns10262 21054140

[32] Woodworth GF Patel KS Shin B Burkhardt JK Tsiouris AJ McCoul ED. Surgical Outcomes Using a Medial-To-Lateral Endonasal Endoscopic Approach to Pituitary Adenomas Invading the Cavernous Sinus. J Neurosurg (2014) 120(5):1086–94. doi: 10.3171/2014.1.jns131228 PMC4249951 24527820

[33] de Paiva Neto MA Vandergrift A Fatemi N Gorgulho AA Desalles AA Cohan P. Endonasal Transsphenoidal Surgery and Multimodality Treatment for Giant Pituitary Adenomas. Clin Endocrinol (2010) 72(4):512–9. doi: 10.1111/j.1365-2265.2009.03665.x 19555365

[34] Gerganov V Metwali H Samii A Fahlbusch R Samii M. Microsurgical Resection of Extensive Craniopharyngiomas Using a Frontolateral Approach: Operative Technique and Outcome. J Neurosurg (2014) 120(2):559–70. doi: 10.3171/2013.9.JNS122133 24266540

[35] Poblete T Jiang X Komune N Matsushima K Rhoton AL Jr. Preservation of the Nerves to the Frontalis Muscle During Pterional Craniotomy. J Neurosurg (2015) 122(6):1274–82. doi: 10.3171/2014.10.JNS142061 25839922

[36] Yamada S Fukuhara N Horiguchi K Yamaguchi-Okada M Nishioka H Takeshita A. Clinicopathological Characteristics and Therapeutic Outcomes in Thyrotropin-Secreting Pituitary Adenomas: A Single-Center Study of 90 Cases. J Neurosurg (2014) 121(6):1462–73. doi: 10.3171/2014.7.jns1471 25237847

